# Emergency pancreatoduodenectomy for non-traumatic conditions: a case series analysis

**DOI:** 10.1186/s12876-025-03868-1

**Published:** 2025-04-26

**Authors:** Xinxiong Li, Weixuan Hong, Zhongdong Zou, Junhao Du, Ruohan Su, Lie Wang, Chunhong Xiao, Meiping Wang

**Affiliations:** 1 Department of General Surgery, Dongfang Hospital of Xiamen University/900 th Hospital of the United Logistics Force, 156 Xierhuan Northern Road, Fuzhou, Fujian 350025 People’s Republic of China; 2 Department of Thyroid Hernia Surgery, Fujian Provincial Hospital, Fuzhou University, Fuzhou, Fujian China

**Keywords:** Non-traumatic, Pancreaticoduodenectomy, Emergency surgery, Etiology

## Abstract

**Background:**

Emergency pancreaticoduodenectomy (EPD) is an uncommon abdominal surgical procedure primarily performed in patients with severe acute abdominal trauma. Performing EPD requires strict surgical criteria, advanced technical expertise, and comprehensive postoperative management. Limited research exists regarding the indications for non-traumatic EPD. Thus, the objective of this study was to synthesize and analyze recent cases of non-traumatic EPD, thereby enhancing the comprehension of this urgent surgical measure.

**Methods:**

A retrospective analysis was conducted on patients who underwent non-traumatic EPD at the 900th Hospital of the Joint Logistics Support Force of the Chinese People’s Liberation Army from January 2013 to September 2023. The study assessed preoperative etiologies, intraoperative findings, postoperative complications, and prognosis. Additionally, a literature review was performed.

**Results:**

Nine patients underwent non-traumatic EPD. All patients demonstrated clear indications for emergency surgery: three cases of gastrointestinal hemorrhage secondary to ulcerative lesions, three cases of perforation (*n* = 2) and bleeding (*n* = 1) following invasive endoscopic procedures for neoplastic lesions, two cases of tumor rupture-related hemorrhage, and one case of postoperative anastomotic bleeding. All patients completed the surgical procedure. The operative duration ranged from 185.0 to 480.0 min, with a mean of 299.9 ± 83.3 min, and intraoperative blood loss ranged from 100.0 to 6,000.0 ml, with a mean of 1,477.8 ± 1,944.7 ml. Postoperative pathology revealed that 3 cases involved benign ulcerative lesions of the digestive tract and 6 cases involved neoplastic lesions in the pancreaticoduodenal region. One patient died 6 days postoperatively due to multiple organ failure, another died 42 days postoperatively due to tumor progression, and the remaining 7 patients recovered and were discharged, with a postoperative hospital stay of 17–45 days, mean 36.3 ± 10.5 days. Postoperative complications occurred in six patients (85.7%), including pancreatic fistula, biliary fistula, and abdominal infection, all of which resolved with conservative management.

**Conclusion:**

In cases of non-traumatic emergencies in the pancreaticoduodenal region where conservative or minimally invasive treatments fail to control the acute progression, EPD serves as a critical surgical intervention that may save lives and yield favorable outcomes.

## Introduction

Since its initial description by Whipple in 1935, pancreaticoduodenectomy (PD) has undergone nearly a century of refinement and has become the gold-standard surgical approach for periampullary pathologies. The anatomy of the pancreatic head-duodenal region is complex, and PD necessitates resection of multiple organs and reconstruction of multiple digestive tracts, which often leads to multiple postoperative complications. Elective PD is already a high-risk procedure, and emergency pancreaticoduodenectomy (EPD) is even more rarely performed clinically, with scant reports of related studies. EPD was initially used for trauma-induced injuries of the pancreatic head or duodenum, often accompanied by visceral hemorrhage and compound injuries of peripheral organs, and is associated with severe complications such as hypovolemia, hypothermia, and coagulation disorders. Serious complications such as hypovolemia, hypothermia, and coagulation disorders present major challenges to emergency resuscitation efforts. [1]. Therefore, EPD necessitates stringent surgical criteria, advanced operative skills, and robust postoperative management. In non-traumatic scenarios, for emergencies due to acute uncontrolled hemorrhage, perforated peritonitis, obstruction, and other lesions in the pancreatic head-duodenal region, EPD may be the last resort for saving the patient's life when aggressive conservative medical treatments, endoscopy, and interventional embolization fail to control disease progression. Thus, This study retrospectively analyzed non-traumatic EPD cases at our institution to guide clinical management and improve patient survival.

## Materials and methods

### General information

PD conducted at the Department of General Surgery, 900 th Hospital of the Joint Logistics Support Force, from January 2013 to September 2023, were retrospectively analyzed in 419 patients. Among these, 12 patients underwent emergency surgery, 3 underwent emergency pancreaticoduodenectomy (EPD) due to trauma, and 9 due to non-traumatic factors. Inclusion criteria included: (1) admission to the emergency department or referral from an external hospital for emergency surgery or in-hospital unplanned emergency procedures; (2) a clear indication for emergency surgery based on imaging and clinical diagnosis; (3) the surgical options include standard PD and PD-modified surgery, including pyloro-sparing pancreaticoduodenectomy (PPPD), vascular resection and reconstruction, and subtotal gastric-preserving PD, and extended PD with resection of other organs.; (4) complete clinical data were available. Exclusion criteria included: (1) patients with evident trauma factors; (2) patients who underwent elective surgery following improvement or stabilization with conservative treatment. The study cohort comprised 8 males and 1 female, with ages ranging from 19 to 81 years and a mean age of 63 ± 19 years. Patients'demographic data, indications for emergency surgery (preoperative etiology), intraoperative conditions, postoperative complications, and survival rates were recorded and analyzed. Written informed consent was obtained from all patients and their families regarding treatment. Clinical data of the operated patients are presented in Table 1.

**Table 1:**
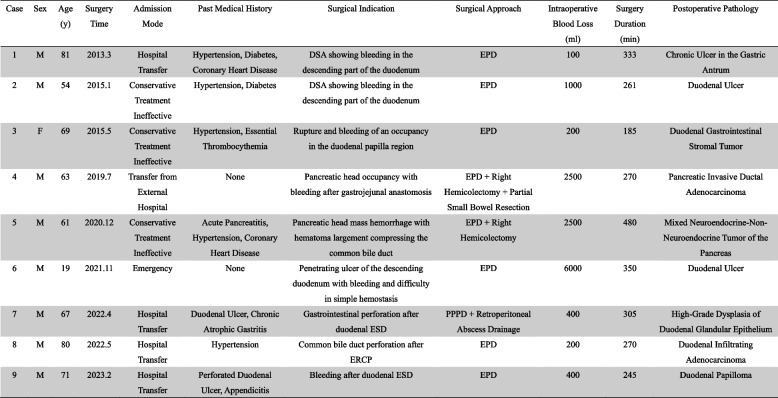
Clinical data of 9 cases of EPD patients

*F *Female, *M *Male, *y *years, *DSA *Digital Subtraction Angiography, *ESD *Endoscopic Submucosal Dissection, *ERCP *Endoscopic Retrograde Cholangiopancreatography, *EPD *Emergency Pancreaticoduodenectomy, *PPPD *Pylorus Preserving Pancreaticoduodenectomy

### Treatment plan

All patients had clear surgical indications based on preoperative examinations and clinical presentations. The surgical plan was confirmed based on preoperative manifestations and intraoperative findings, with at least two attending physicians with extensive clinical experience making the determination prior to conducting EPD. The lead surgeons had more than 30 years of clinical experience in pancreaticoduodenal surgery, and the team members also had deep attainments and rich practical experience in the field of pancreatic surgery. Routinely, the surgically excised specimens underwent pathological testing, with the review and confirmation of pathological sections conducted by two independent pathologists. After surgery, all patients were admitted to the intensive care unit for close monitoring and treatment. Follow-up for surviving patients was conducted through outpatient visits or telephone contact.

## Results

### Timing of surgery

**Fig. 1:**
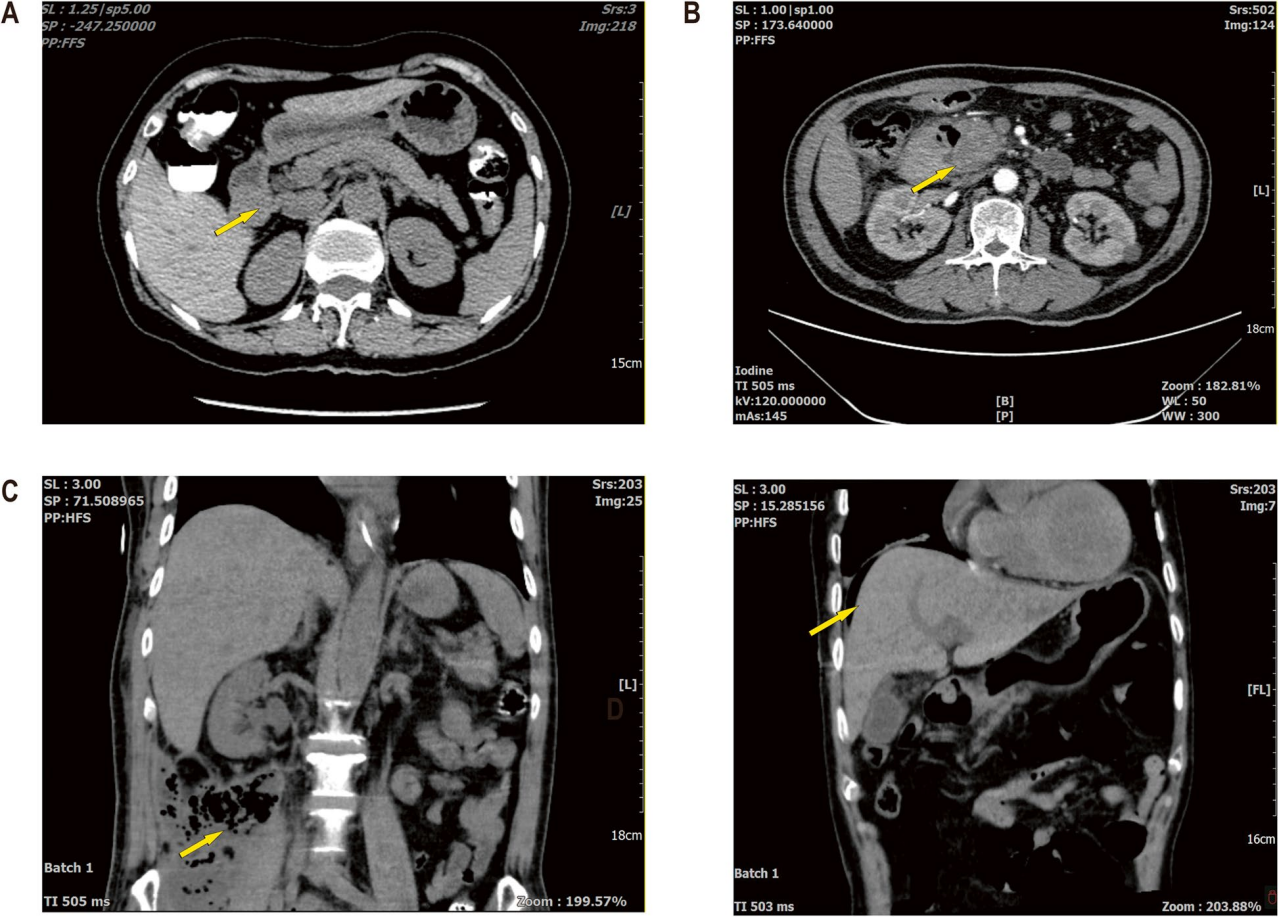
Preoperative imaging examination of some patients. **A** Case 3: Occupying the duodenal bulb. **B** Case 5: Occupying the head of the pancreas. **C** Case 7: Presenting with an abdominopelvic infection focus. **D** Case 8: Showing free air below the diaphragm following ERCP stent placement

**Fig. 2:**
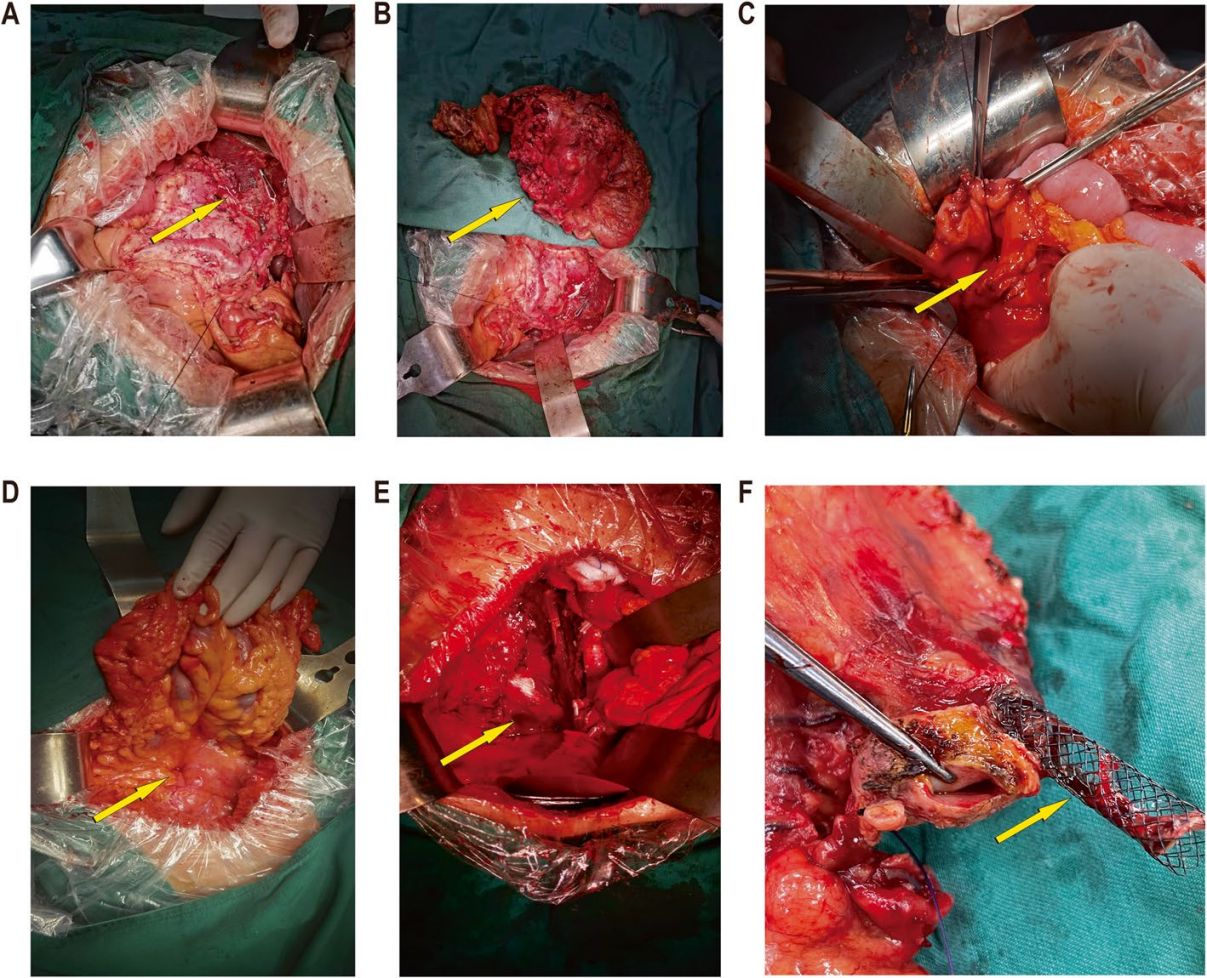
Displays the intraoperative conditions of some patients. **A** Case 5: Where the abdominal cavity was explored and a huge tumor with bleeding was revealed. **B** Case 5: Where the tumor was completely removed using EPD. **C** Case 6: Where the duodenal ulcer presented difficulty in achieving hemostasis. **D** Case 7: Where exploration revealed a duodenal perforation. **E** Case 7: Where exploration revealed a retroperitoneal abscess cavity. **F** Case 8: Where a perforation was observed following ERCP stent placement

All patients received conservative medical treatment or minimally invasive procedures prior to surgery, which were ineffective, and had definite indications for emergency surgery. Case 1: Digital Subtraction Angiography (DSA) failed to achieve hemostasis, a 2 × 3 cm solid mass was palpable in the descending part of the duodenum with inflammatory infiltration of the surrounding tissues, indistinct margins, and overt bleeding. Case 2: DSA was ineffective in controlling bleeding; a significant hematoma was found in the gastric, and a 3 × 3 cm solid mass was palpable in the descending part of the duodenum with inflammatory infiltration of the surrounding tissues and indistinct margins. Case 3: A 2 × 3 cm solid mass was observed in the duodenal papilla region, infiltrating the serosal layer and associated with bleeding. Case 4: Postoperative alterations following gastrojejunal anastomosis at an external facility, hemoperitoneum with blood clots in the abdominal cavity, a mass of about 2 × 3 cm was palpable in the duodenal pancreatic head region, and a substantial quantity of dark red blood and clots were drained from the gastric. Case 5: An gaint mass measuring about 10 × 10 cm in the pancreatic head and duodenal area, with internal hemorrhage and compression of the common bile duct due to the hematoma. Case 6: Active bleeding from an ulcer in the descending duodenum was noted, characterized by a firm consistency and irregular ulcerated mucosa; endoscopic attempts at hemostasis were futile, and intraoperative incision and suturing for hemostasis proved difficult. Case 7: Post-Endoscopic Submucosal Dissection (ESD) gastrointestinal perforation was unresponsive to conservative management; upon exploration, retroperitoneal infection was found, along with edema of the hepatic flexure and ascending colon, and inflammatory adhesions surrounding the horizontal segment of the descending duodenum. Case 8: Post-Endoscopic Retrograde Cholangiopancreatography(ERCP), perforation of the common bile duct was identified; the exploration showed a dilated common bile duct with a stent traversing parallel to the lateral wall in the lower part, resulting in a dissection. Case 9: Post-duodenal ESD, perforation and hemorrhage were observed; a 1 × 1 cm perforation was identified on the anterior and medial walls of the descending duodenum. The preoperative imaging studies of some patients are depicted in Fig. 1, and the intraoperative findings of some patients are illustrated in Fig. 2.

### Surgical approach

All patients successfully completed the surgery. The standard procedure involved resection of the distal gastric, duodenum, proximal jejunum, pancreatic head, common bile duct, and gallbladder. Case 4 underwent an extended PD because of tumor involvement of the transverse colon and small intestine, including a right hemicolectomy and partial small bowel resection. Case 5 underwent an extended PD because of tumor involvement of the middle colic vessels, including a right hemicolectomy. Case 6 underwent combined intraoperative endoscopic hemostasis and duodenotomy with suture ligation for hemostasis. Case 7 underwent a pylorus-preserving pancreaticoduodenectomy and had an incision and drainage of a retroperitoneal abscess.

For gastrointestinal reconstruction, Case 6 employed a pancreaticojejunal invaginated anastomosis due to challenging intraoperative pancreatic duct exploration, while other cases utilized pancreatic duct-to-mucosa anastomosis with stent placement (Blumgart's method). The standard procedure included choledochojejunal end-to-side anastomosis and gastrointestinal Roux-en-Y anastomosis. Each patient had a single Jackson-Pratt drain placed near the pancreaticojejunal and choledochojejunal anastomoses, with Cases 4–9 receiving additional peritoneal drains posterior to the pancreaticojejunal anastomosis and along the gastrointestinal anastomosis, and Case 7 receiving an additional Jackson-Pratt drain in the retroperitoneal abscess cavity. The surgical duration varied from 185.0 to 480.0 min, averaging 299.9 ± 83.3 min, and intraoperative blood loss ranged from 100.0 to 6000.0 ml, averaging 1477.8 ± 1944.7 ml.

### Postoperative pathology

**Fig. 3:**
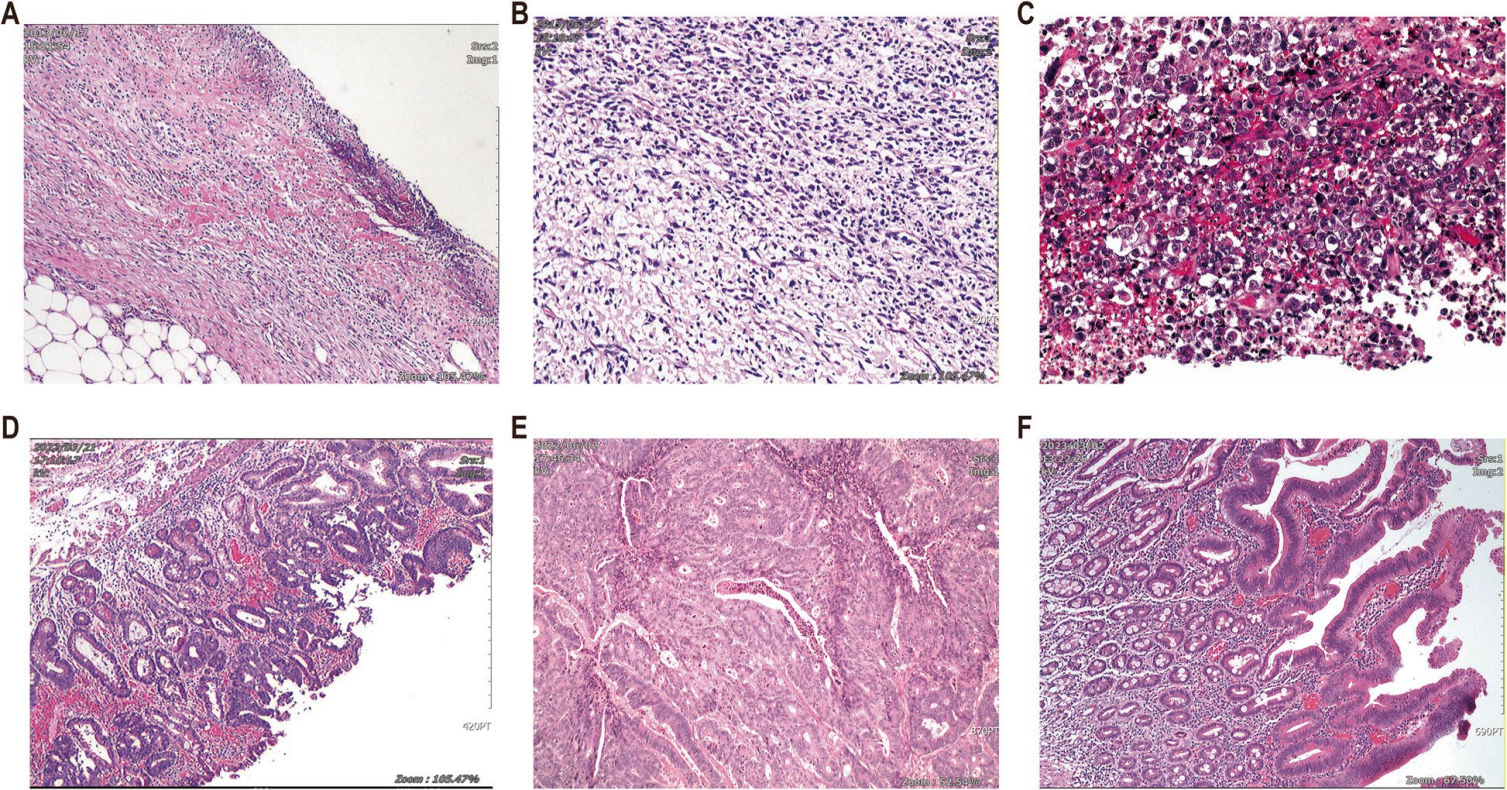
Pathological results of some patients. **A** Case 1: Chronic gastric ulcer. **B** Case 3: Duodenal Gastrointestinal Stromal Tumor. **C** Case 5: Pancreatic mixed neuroendocrine-non-neuroendocrine tumor. **D** Case 7: High-Grade Dysplasia of Duodenal Glandular Epithelium. **E** Case 8: Duodenal Infiltrating Adenocarcinoma. **F** Case 9: Duodenal Papilloma

Pathological examination after complete removal of tissues showed that three patients had ulcerative lesions in the gastrointestinal tract, including two duodenal ulcers and one ulcer in the gastric antrum; six patients had neoplastic lesions, including four duodenal tumors: duodenal gastrointestinal stromal tumor, high-grade dysplasia of duodenal glandular epithelium, duodenal infiltrating adenocarcinoma, and duodenal papilloma; and two cases of pancreatic tumors, one case of pancreatic invasive ductal adenocarcinoma, and a rare mixed neuroendocrine-non-neuroendocrine tumor of the pancreas [2]. The pathologic findings of some patients are shown in Fig. 3.

### Postoperative complications and prognosis

All patients were admitted to the intensive care unit (ICU) for further treatment after surgery, where their vital signs were maintained, hematological indicators were dynamically rechecked, abdominal organ functions were assessed, and treatment was managed with integrated traditional Chinese and Western medicine. Additionally, timely warnings and interventions were implemented for suspected complications. Postoperative recovery and prognosis of EPD are shown in Table 2. Case 4 expired 6 days postoperatively due to the development of irreversible multiorgan failure. Case 5 experienced leukocytosis and hypercalcemia 3 weeks postoperatively, and a multidisciplinary consultation was conducted to assess a leukemia-like reaction potentially caused by malignant tumors, and expired due to progressive disease at 42 days postoperatively. 2 patients with non-traumatic EPD died perioperatively in our center.. The remaining 7 patients recovered and were discharged from the hospital, with postoperative ICU monitoring time of 5–14 days, mean 8.5 ± 3.0 days. Postoperative hospitalization time was 17–45 days, mean 36.3 ± 10.5 days.

**Table 2:**
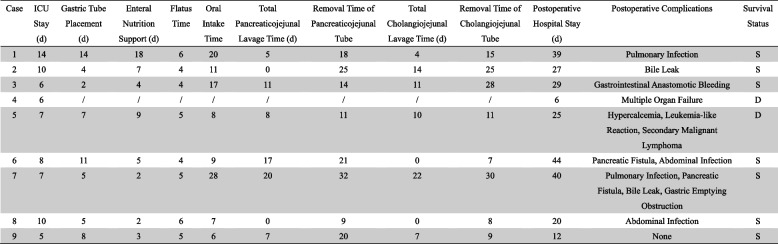
Postoperative recovery and prognosis of 9 EPD patients

*d *days, *S *Survived, *D *Deceased

Postoperative complications were observed in 6 patients, with an incidence rate of 85.7%, of which 3 cases combined multiple complications. The complications included pancreatic fistula in 2 cases, both of which were grade B pancreatic fistula; biliary fistula in 2 cases, both grade A biliary fistula;abdominal infection in 2 cases; pulmonary infection in 2 cases; gastrointestinal anastomosis bleeding in 1 case, which was grade B postoperative pancreatic bleeding; and gastric emptying obstruction in 1 case. The patients with pancreatic fistula, biliary fistula, and abdominal infection were treated with symptomatic treatments such as irrigation and drainage, nutritional support, and infection control. The patients with pulmonary infection were treated with symptomatic treatments such as respiratory support and infection control. The patient with gastrointestinal anastomotic bleeding was treated with emergency endoscopic hemostasis. The patient with gastric emptying obstruction was treated with gastroprokinetic drugs, enteral nutrition, acupuncture, and physiotherapy. All of them were cured and discharged from hospitals after conservative treatments, and there were no patients who underwent secondary surgeries. As of the follow-up in September 2024, the median follow-up time was 34 months. Among them, Patient 1 died naturally 4 years after follow-up, the remaining 6 patients were in good condition, and 2 had chronic symptoms such as postoperative dyspepsia and dependence on pancreatic enzyme replacement therapy.

## Discussion

With the improvement of surgical safety and the implementation of standardized postoperative treatment, the incidence of postoperative complications and mortality of PD has decreased significantly [3, 4]. Compared with elective PD, non-traumatic EPD lacks adequate preoperative preparation and selection of optimal treatment regimens, and rapid changes in the condition require more stringent operation and postoperative management by the operator [5]. Currently, the incidence of non-traumatic EPD in different centers reaches 1–2% [6–8].There are limited cases of EPD research currently, and according to the latest research, the in-hospital mortality rate of EPD caused by bleeding is 9.38% [9], but in the past, the mortality rate of EPD was as high as 17%− 40%. The incidence of non-traumatic EPD in our center is 2.1%, and the mortality rate is 22.2%, and the overall postoperative complication rate and mortality rate of EPD are higher than that of elective PD [10, 11]. To improve patient survival, it is crucial to clarify the surgical indications for EPD and select the optimal timing of surgery. Therefore, we analyzed and summarized the preoperative etiology of non-traumatic EPD based on relevant literature reports and our center's treatment experience.

### Bleeding

Uncontrollable bleeding is the most common etiology in all patients undergoing EPD. Acute hemorrhage in the pancreaticoduodenal area is usually caused by ulcerative bleeding or neoplastic lesions eroding the blood vessels, and rare cases have been reported of specific lesions such as duodenal Dieulafoy lesions [12], arteriovenous malformation around the head of the pancreas [13], and diffuse large B-cell lymphoma [14]. In our case reports, up to 77.8% of cases underwent EPD for bleeding. Some bleeding can be stabilized by endoscopic and radiological interventions to stabilize the patient's vital signs and achieve hemostasis [15], but this treatment has some limitations. In case 6, despite several attempts to stop bleeding with endoscopic hemostatic clips, it was not possible to control the active bleeding in the deep mucosa, and the intraoperative exploration and incision of the duodenum revealed a hard ulcerated surface and uneven mucosa, which made it difficult to stop the bleeding with sutures, so PD was finally chosen. In the case of internal bleeding caused by advanced tumors, the site of bleeding is difficult to determine, and the tumors may also compress the gastrointestinal tract, which may affect endoscopic examination and manipulation [14].In case 3, the tumor was located at the duodenal bulboduodenal junction, and considering the special location of the tumor and the fact that it was partially located outside the lumen, endoscopic surgery was risky and difficult to control bleeding. In addition, because the pancreaticoduodenal region has strong collateral circulation from the abdominal cavity and superior mesenteric artery, and the tumor has a rich blood supply, vascular embolization alone may not be able to effectively control hemorrhage [16].In case 5, arterial embolization was performed after considering tumor hemorrhage, but a follow-up Computed tomography(CT) scan showed that the hematoma area around the head of the pancreas was further expanding and even compressing the common bile duct. Of course, a combination of less invasive therapies could temporarily stabilize the patient's hemodynamics while emergency surgery was pursued. Surgical exploration focuses on early and rapid control of the peripancreatic vascular system, including the superior mesenteric artery, portal vein, and pancreaticoduodenal vascular arch [17], followed by PD to achieve the dual effect of relieving the primary tumor lesion and managing complications.

### Piercing

The pancreaticoduodenal region can be perforated by ulcerative lesions, neoplastic lesions, and medical injury. The duodenum receives bile, pancreatic fluid, gastric fluid, and food residue, and the condition progresses rapidly after perforation, and in severe cases can develop into acute peritonitis or even infectious shock. The focus of emergency surgery is to identify and localize the site of injury to the duodenum. When exploration reveals a large perforated ulcer (> 2 cm), the surrounding tubular wall tends to be more fragile, making simple repair or anastomosis difficult, and there is a risk of postoperative leakage [7, 18]. Previous cases have reported patients who had to undergo EPD due to peritonitis complicating suture splitting after duodenal repair [16]. In addition, when the perforation site is not in the duodenal descending portion, pancreas-sparing duodenectomy can be tried [19]. Another focus of perforation surgery is to explore the abdominal cavity for the detection of infected foci and to perform adequate debridement and drainage. In case 7, CT after perforation showed free gas in the abdominal cavity, secondary peritonitis was still present after aggressive conservative treatment, and CT review showed fluid and gas accumulation in the abdominopelvic cavity and retroperitoneum, which progressed from the previous case. Retroperitoneal abscesses caused by duodenal perforation are relatively rare [20, 21], which is difficult to recognize and localize when the perforation is located in the posterior duodenum or retroperitoneum. When opening the posterior peritoneum to remove the effusion, careful blunt separation of the entire C-shaped duodenum is required due to the presence of adhesions around the bowel to prevent duodenal laceration or further enlargement of the injury [22]. Placement of an abdominal drain for patency and drainage is required after debridement to minimize the risk of postoperative infection.

### Medically induced injuries

With the improvement of endoscopic instruments, technology, and theoretical discipline, endoscopic diagnosis and treatment technology has developed from the stage of pure diagnosis to the advanced stage of minimally invasive intervention integrating diagnosis and treatment. However, some serious complications still cannot be avoided, and medical-related injuries have been an important etiology of emergency surgical interventions in recent years. An incidence of ERCP perforation reaches 0.2% [23]. Risk factors for perforation include advanced age of the patient, sphincter of Oddi dysfunction, bile duct dilatation, prolonged duration of the procedure, sphincterotomy, and stent placement [6, 24]. When perforation is suspected, close monitoring of the patient's clinical symptoms and peritoneal signs and prompt abdominal CT are required. Small amounts of free gas can be treated conservatively, including fasting, use of proton pump inhibitors, anti-infection, endoscopic nasobiliary drainage, and application of metal clips or bioadhesive blockage. In case 8, abdominal pain and fever, abdominal muscle tension with pressure and rebound pain, and elevated inflammatory indexes were observed following ERCP. Therefore, the finding of a significant increase in infected fluid or typical signs of peritoneal irritation indicates that bile and pancreatic fluid are still continuing to leak out [25], indicating that a laparotomy is urgently required.

Currently, ESD has become an effective strategy for the treatment of duodenal tumors, and serious complications accompanying ESD include delayed perforation and bleeding. According to results from other research centers, the rate of intraoperative perforation with ESD ranges from 6.3% to 7.5%, and the rate of delayed perforation ranges from 0% to 14.3% [26]. The narrow and curved lumen of the duodenal region tends to result in a restricted endoscopic field of view, and the abundant submucosal and thin muscular layer of blood vessels, as well as a weaker muscular layer relative to the rest of the GI tract [27]. These special anatomical structures make the risk of perforation and hemorrhage higher. In addition, the size of the tumor is one of the important risk factors for delayed bleeding [28, 29]. Case 9 underwent ESD for a duodenal papillary tumor, and preoperative evaluation showed that the tumor size was about 2.5 × 3.5 cm, and bleeding was repeated during endoscopic tumor debridement, and after hemostatic forceps were used to treat the wound, the muscularis propria was seen to be broken, and considering the large size of the wound and its complex location, endoscopic treatment became difficult to convert to surgical intervention. Therefore, before endoscopic treatment, the patient's risk factors should be fully evaluated, and when complications occur and endoscopic perforation sealing or hemostasis is ineffective, emergency surgery is an important guarantee to save the condition.

### Necrosis or ischemia

Diseases related to ischemia and necrosis have been reported as indications for surgery for EPDs, including duodenal necrosis [30], necrotizing pancreatitis [16], necrotizing cholecystitis combined with choledochal necrosis, and others. Pancreatitis may be an important factor contributing to duodenal necrosis. The release of pancreatic enzymes and changes in the inflammatory response lead to vascular injury and arterial thrombosis, which in turn develops into transmural necrosis of the duodenum, a lesion that occurs predominantly in the descending and horizontal portions of the duodenum [30–32].Due to the unique blood supply and tissue structure of the duodenum, necrosis of the duodenum is still relatively rare. Intraoperative exploration does not recommend simple partial resection and anastomosis of the duodenum, depending on the extent of necrotic tissue, but requires complete pancreaticoduodenectomy [33]. In addition, duodenal intussusception [34], non-metastatic tumors invading the head of the pancreas, and obstruction-related diseases such as extensive tumors in the duodenum are equally amenable to EPD. In conclusion, it is crucial to define the surgical indications and the timing of salvage for EPD, which directly affects intraoperative manipulation, the occurrence of postoperative complications, and the prognosis.

Phase I GI reconstruction was completed in all cases. According to our center's treatment experience, the key points of EPD intraoperative operation are summarized: (1) focus on early control of peripancreatic vasculature in patients with high-risk hemorrhage; (2) assess the impact of pancreatic tenderness and intraoperative blood loss on the risk of postoperative pancreatic fistula; (3) rationally select the pancreatico-enteric anastomosis; for the pancreatic ductal stumps that are difficult to explore intraoperatively, pancreatico-jejunal sleeve anastomoses are feasible; for pancreatic ductal dilatation, Blumgart's anastomosis is indicated; (4) severe abdominal infections combined with perforation should be fully explored, cleared, and drained intraoperatively; (5) pancreatic and biliary drainage tubes are routinely placed in the postoperative period, and abdominal drainage tubes can be added in the posterior part of pancreatic-enteric anastomosis and gastrointestinal anastomosis. In addition, other centers proposed damage control PD [35, 36]. Damage control surgery (DCS) was initially applied to abdominal trauma, and it can effectively reduce morbidity, mortality, and complications through the stages of rapid surgical control of the injury, ICU resuscitation, and definitive reoperation [37, 38]. Considering the patient's potentially poor baseline condition and preoperative instability, high inflammatory reaction in the intraoperative exploration of the operative area, edema of the surrounding tissues, and high failure rate of anastomotic reconstruction, and prolongation of radical treatment surgery and surgical injuries further aggravate the blow to the patient. Non-invasive DCS utilizes a two-stage treatment strategy that involves first relieving potentially fatal complications followed by radical repair and reconstruction [11]. Case 4 was referred to our institution for resuscitative treatment with EPD due to hemorrhagic shock after initial surgery at an outside institution, and died 6 days postoperatively due to multiorgan failure. Multiple surgical trauma coupled with malignant consumption caused by primary pancreatic malignancy, damage control PD may be more suitable for this type of critically ill patients. Of course, the choice of surgical plan should be determined by the judgment of an experienced operator based on preoperative status and intraoperative changes in condition.

The incidence of postoperative complications after EPD is as high as 80–90% [8]. Among them, pancreatic fistula is the most common postoperative complication. The occurrence of pancreatic fistula is associated with the advanced age of the patient, soft pancreatic texture, small diameter of the pancreatic duct, and intraoperative blood loss, etc. Pancreatic fistula usually leads to complications such as abdominal infection, postoperative bleeding, and delayed gastric emptying. Most pancreatic fistulas can be treated conservatively through aggressive patency drainage and infection control methods [31, 39]. Postoperative bleeding is often more critical and includes gastrointestinal bleeding and intra-abdominal bleeding. Gastrointestinal bleeding can be treated by endoscopic techniques, based on the resection of the leptomeningeal mesentery, the exact suture around the portal vein and abdominal aorta to stop hemorrhage, and the gastroduodenal arterial stump can be effectively prevented from intra-abdominal hemorrhage by the ligature and then suture. When there is a significant hemodynamic instability or a high degree of suspicion of hemorrhage due to a severe intra-abdominal infection, anastomotic leakage, or erosion of blood vessels, and then timely dissection is carried out to investigate the hemorrhage.

Our study also has certain limitations. The retrospective analysis has a relatively small sample size and lacks a control group comparison with elective PD to further clarify the differences in efficacy and safety between emergency surgery and planned surgery. In the future, we hope to establish a standardized multicenter control cohort, further expand the sample size, optimize case selection, and enhance the persuasiveness of the research results.

## Conclusion

In summary, EPD is a rare but effective clinical treatment for non-traumatized patients. Morbidity and mortality depend on the clinical status of the patient, the experience of the surgeon, and the level of the medical treatment center. Based on the patient's clinical presentation and examination findings, EPD as an urgent surgical intervention can save the patient's life and achieve a good prognosis when conservative or less-invasive treatments fail to control the progression of the acute condition, but the indications for surgery need to be carefully considered.

## Data Availability

The datasets used during the current study are available from the corresponding author on reasonable request.

## Abbreviations

EPD: Emergency Pancreaticoduodenectomy
PD: Pancreaticoduodenectomy
PPPD: Pylorus Preserving Pancreaticoduodenectomy
DSA: Digital Subtraction Angiography
ESD: Endoscopic Submucosal Dissection
ERCP: Endoscopic Retrograde Cholangiopancreatography
CT: Computed tomography
ICU: Intensive Care Unit
DCS: Damage control surgery
